# Unveiling the functional nature of retrogenes in dinoflagellates

**DOI:** 10.1098/rsob.240221

**Published:** 2025-04-23

**Authors:** Ronie Haro, Renny Lee, Claudio H. Slamovits

**Affiliations:** ^1^ Department of Biochemistry and Molecular Biology, Dalhousie University, Halifax, Nova Scotia, Canada

**Keywords:** retrogenes, dinoflagellates, spliced-leader, trans-splicing, gene retroposition, mRNA processing, symbiosis

## Abstract

Retroposition is a gene duplication mechanism that uses RNA molecules as intermediaries to generate new gene copies. Dinoflagellates are proposed as an ideal model for exploring this process due to the tagging of retrogenes with DNA-encoded remnants of the dinoflagellate-specific splice-leader motif at their 5′ end. We conducted a comprehensive search for retrogenes in dinoflagellate transcriptomes to uncover their functional nature and the processes underlying their redundancy. We obtained a high-confidence set of hypothetical functional retrogenes widespread through the dinoflagellate lineage. Through annotations and gene ontology enrichment analysis, we found that the functional diversity of retrogenes reflects the most prevalent and active processes during stress periods, particularly those involving post-translational modifications and cell signalling pathways. Additionally, the significant presence of retrogenes linked to specific biological processes involved in symbiosis and toxin production underscores the role of retrogenes in adaptation. The expression profile and codon composition similar to protein-coding genes confirm the operational status of retrogenes and strengthen the idea that retrogenes recapitulate parental gene expression and function. This study provides new evidence supporting widespread gene retroposition across dinoflagellates and highlights the functional link of retrogenes with the core activity of the cell.

**Figure:**
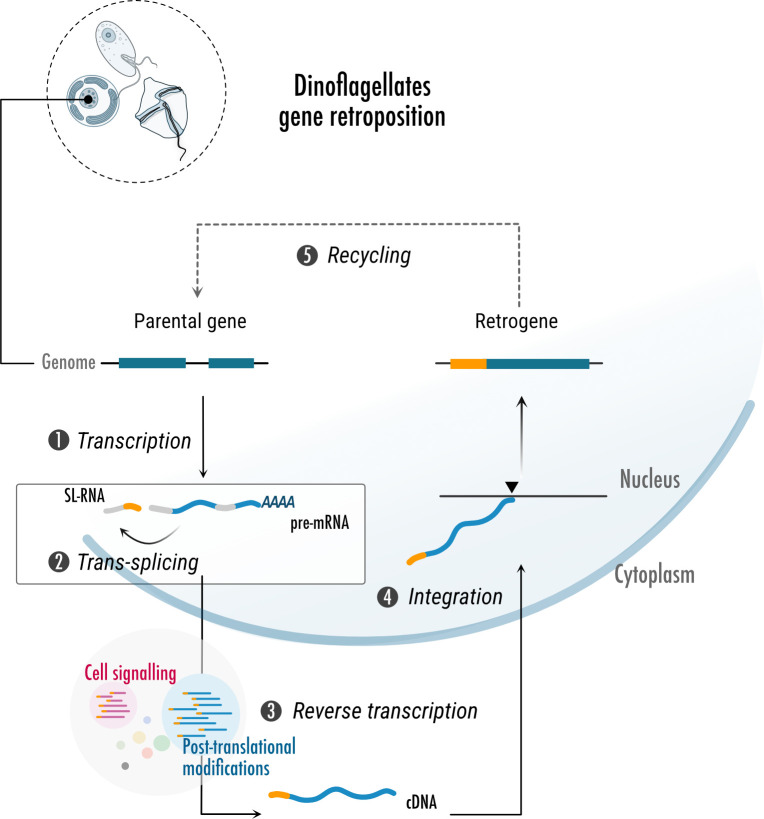

## 1. Introduction

Generating new genes significantly shapes molecular evolution, providing the raw material for the origination of evolutionary novelties [1]. While well-documented DNA-based mechanisms like unequal crossing-over and segmental duplication are widely recognized as the main forces behind gene duplication [2], less-explored RNA-based mechanisms (i.e. retroduplication) can also generate gene duplicates [3]. This type of duplication demands the off-target activity of reverse transcriptase derived from retrotransposons acting on the host mRNA [4]. Notably, LINE1 retrotransposon activity has been demonstrated to create retroposed gene copies in mammalian cell lines [5,6]. In retroduplication, the mRNA of a parent gene undergoes reverse transcription and is subsequently integrated into a new genomic locus [3]. The resultant duplicate consists of only exons devoid of *cis*-regulatory elements (e.g. promoters). Most retrocopies are non-functional (dead upon arrival) as they lack a promoter and lose their coding potential due to the accumulation of frameshift mutations causing premature stop codons [7]. However, retrocopies can sometimes escape this erosion, turning into *bona fide* genes (i.e. retrogenes) [8].

Furthermore, it has been shown that retrogene functionality highly depends on the recruitment of regulatory sequences. They can be acquired from the new genomic neighbourhood or its retrotransposon mediator (reviewed in [3]). As a result, retrogenes are prone to develop novel expression patterns that lead to new evolutionary trajectories and roles [9,10]. In contrast, segmental duplication produces gene copies that primarily mirror the parental function. There is growing evidence supporting retrogene participation in a variety of processes, including subcellular relocalization of proteins, neurotransmission [11], tumour development [12] and antiviral defence [13]. Additionally, since it is not only protein-coding transcripts that can become substrates for retrotranscription, some retrogenes have regulatory functions as non-coding RNA. Retrogene formation represents a mechanism of gene emergence from *a priori* non-functional sequences and the recruitment of gene regulatory sequences from scratch. Nevertheless, understanding it is challenging, and surveying retrogenes in less-well-studied organisms may provide additional insight.

Retrogene research has predominantly focused on model organisms with an emphasis on mammals and *Drosophila melanogaster* because of the retention of young retrogenes [14–17]. In contrast, there has been relatively less exploration of less-studied lineages such as green algae [18] and dinoflagellates [19–21]. Retrogenes are particularly abundant in the latter, but little is known about their persistence and significance for adaptation and genome evolution. In dinoflagellates, identification of retrogenes is facilitated by the presence, at the DNA level, of a partial dinoflagellate-specific spliced-leader motif (DinoSL) upstream of the coding sequence. This DinoSL results from the *trans*-splicing of a short approximately 22-nucleotide SL RNA to the 5′ end of the pre-mRNA of all protein-coding genes [20,22], a process termed spliced leader *trans*-splicing (SLTS) that occurs in a handful of eukaryotic lineages [23]. Since retrogenes originate by reverse transcription of DinoSL-containing messenger RNA followed by insertion in the genome, transcripts from retrogenes will exhibit a fragment of the parental gene’s DinoSL (termed DinoRL) immediately after the leader attached by SLTS. Such relic has been used as a ‘tag’ to facilitate retrogene identification from expressed sequence tag (EST) data [19,20] and genomic sequence [21]. Retrogenes are abundant, accounting for 22–25% of the total genes in *Symbiodinium* genomes [21]. Two massive retroposition episodes were inferred for *Breviolum minutum* and *Symbiodinium kawagutii,* leading to the enrichment of retroposed genes related to ion and transmembrane transport, photosynthesis and symbiosis establishment [21]. These retrogenes have been proposed as crucial for adapting to symbiotic life. Interestingly, the abundance of particular retrogenes may correlate with the expression level of their parental genes, meaning that highly expressed genes have higher chances of being the target of retroposition [24]. This may explain the accumulation of genes involved in stress response in *Symbiodinium* genomes stimulated by dramatic climate changes [25]. The idea that highly expressed genes become retrogenes is a ‘self-reinforcing model of molecular evolution’, although further corroboration is needed. Retroposition in dinoflagellates may be mediated by retrotransposons found particularly abundantly in several species [26], but retroviruses cannot be ruled out. In terms of functionality, it is unclear how retrogenes persist and become functional. Transcriptional regulation in dinoflagellates is still poorly understood, but dinoflagellates appear to rely on fewer and simpler transcriptional regulatory elements compared with other eukaryotes [27,28]. An in-depth analysis of the presence and distribution of retrogenes in a wide range of dinoflagellates may help understand gene emergence and its diversity. The presence of DinoSL in retrogenes makes the search straightforward, even allowing the identification of retrogenes in intron-containing genes that are otherwise overlooked in other organisms.

Here, we analyse RNA sequencing data and transcriptome assemblies for 45 dinoflagellate species to conduct functional categorization, expression quantification and codon usage analysis on dinoflagellate retrogenes. Our findings show that most retrogenes originate from genes involved in essential cellular communication and environmental response processes, among other well-conserved core activities such as photosynthesis. Retrogene expression and codon bias trends suggest that retrocopies easily become functional retrogenes. The prevalence of retrogenes across dinoflagellates can be associated with pervasive retrotransposon activity.

## 2. Material and methods

### 2.1. Data collection and retrogene identification

Transcriptomes analysed in this study were generated by the Marine Microbial Eukaryote Transcriptome Sequencing Project [29] and further curated [30]. Additional high-quality transcriptome assemblies of the Symbiodiniaceae family [31–36] and *Pyrocystis lunula* [37] were included (electronic supplementary material, table S1). All transcripts shorter than 200 bp were removed. Assembly completeness was assessed using Benchmarking Universal Single-Copy Orthologs, BUSCO (v. 3.0.0) [38], using alveolate_odb10 (171 orthologues). We took advantage of the ubiquitous DinoSL (DCCGTAGCCATTTTGGCTCAAG, D: A, G, T) to find potential retrogenes. DinoRL is a partial relic of DinoSL, with the first seven nucleotides spliced out by a newly added DinoSL. DinoRL originated when transcripts with an attached DinoSL were integrated back into the genome, expressed and subsequently *trans*-spliced with a new DinoSL. Therefore, retrogenes are defined by the presence of DinoRL. Transcripts having at least one DinoRL (i.e. CCATTTTGGCTCAAG) [20] along with DinoSL at their 5′ ends were targeted as potential retrogenes. Retrogene sequences were identified with seqkit [39], using the command seqkit grep (-s -m 5 -i). Retrogene redundancy was reduced by collapsing similar copies within species using CD-HIT (4.8.1, word size −8 and 90% identity) [40], keeping the largest isoform as representative.

### 2.2. Functional annotation

Hypothetical retrogenes were translated into amino acid sequences with TransDecoder v. 5.5.0 (https://github.com/TransDecoder), and proteins were searched against the NCBI non-redundant database using diamond BLASTp 2.12.0 (e-value 1 × 10^−5^, -max-target-seq 1). Additionally, proteins were annotated using InterProScan 5.52−86.0 [41], and PFAM domains were identified using HMMSCAN (HMMER v. 3.1b2) [42] with an e-value cut-off of 1 × 10^−3^. The amino acid sequences inferred from the retrogenes were also queried against our local PANTHER 14.0 database [43] with an e-value cut-off of 1× 10^−3^. Gene Ontology (GO) terms were retrieved for the PFAM domains using the function *bitr* implemented in clusterProfiler (v. 4.0) [44] using *Saccharomyces cerevisiae* as a reference organism.

### 2.3. Enrichment analysis

Enrichment analysis was conducted for dinoflagellates with higher retrogenes counts and transcriptome completeness (BUSCO > 80%; *Gymnodinium catenatum, Alexandrium molinatum* and *Brandtondinium nutricula*). The retrogenes PFAM domain annotation was tested for enrichment against their respective transcriptomes. Significance was determined using Fisher’s exact test, and *p*-values were corrected for multiple comparisons using the Benjamini–Hochberg method. The GO enrichment was conducted in clusterProfiler v. 4.0 [44] using the function enrichGO. GO terms with a *p*-value less than 0.01 were considered enriched. Redundant GO terms were removed using the function simplify (cut-off = 0.6) in clusterProfiler. For enrichment, correlation between retrogenes and genes with high expression profiles simplify cut-off = 0.7 was used. We visualized the results using the function geom_point in the R package ggplot2 (3.3.5) [45].

### 2.4. Expression estimation

The raw reads (SRA) for *G. catenatum* (SRR1296705), *B. nutricula* (SRR1300537) and *A. molinatum* (SRR1296895, SRR1296896, SRR1296897 and SRR1296898) were downloaded from NCBI under BioProject PRJNA231566 [29]. Reads were trimmed using the Trimmomatic v. 0.39 software [46] with a conservative setting [47]. Reads mapping and quantification were conducted by bowtie2 v. 2.4.5 [48] and RSEM software v. 1.3.0 [49]. The relative expression was normalized in transcripts per millions (TPM), normalizing the read count for the gene length divided by a million (scaling factor). Retrogenes and protein-coding CDS with TPM > 1 were retained. The Wilcoxon rank-sum test evaluated the comparison between retrogenes and protein-coding transcript expression levels.

### 2.5. Codon usage analysis

Codon usage indicators GC3s, GC content, the effective number of codons and relative synonymous codon usage values were estimated for hypothetical retrogenes and protein-coding CDS sequences using CodonW v. 1.4.4 software (http://codonw.sourceforge.net). Plots were generated in R using the function ggscatter of ggpubr v. 0.4.0. These estimations were conducted for *G. catenatum*, *A. molinatum* and *B. nutricula*.

## 3. Results

### 3.1. Structure and distribution of retrogenes across dinoflagellate orders

**Figure 1.**
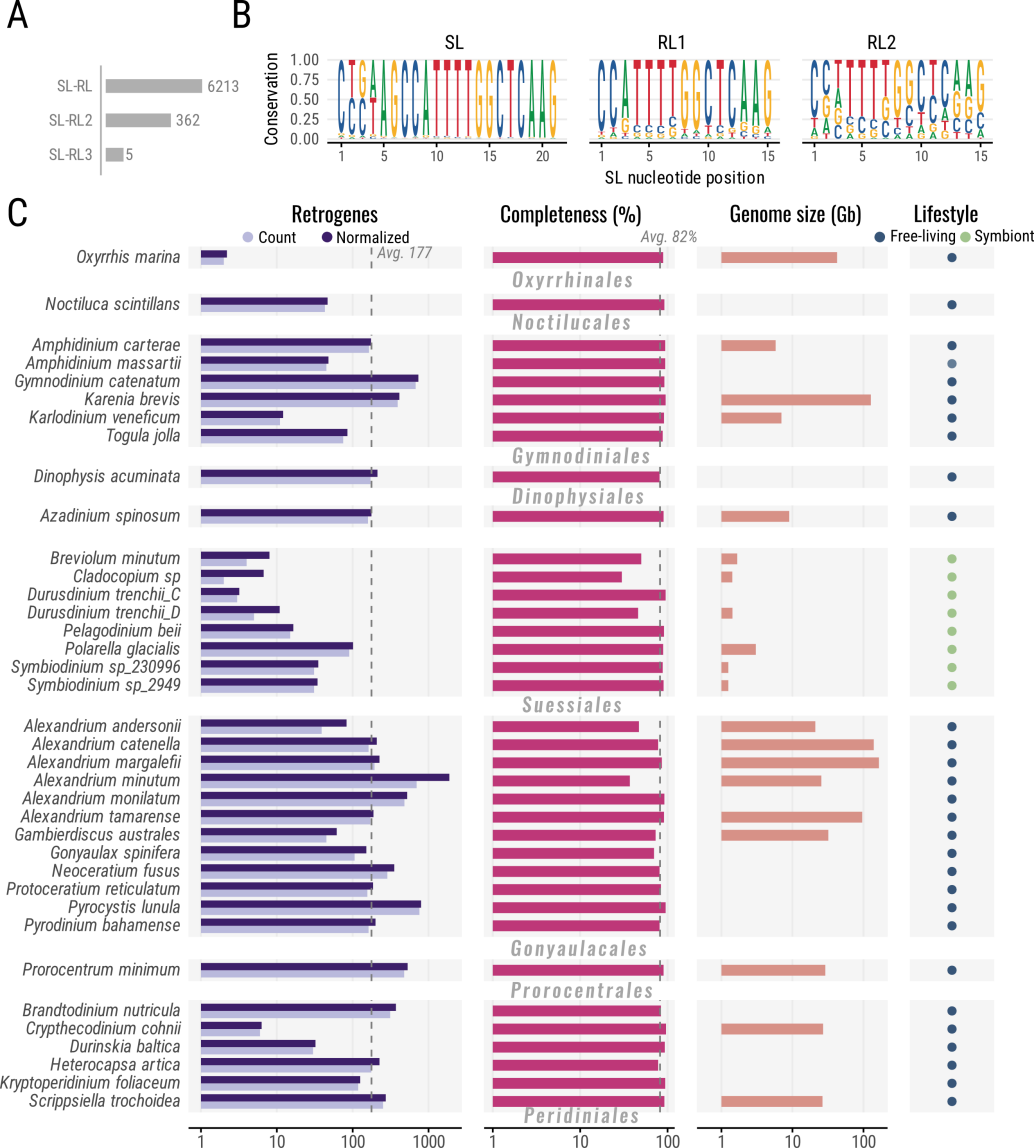
Retrogene survey in dinoflagellate transcriptome assemblies. (A) Number and type of DinoSL/DinoRL arrangements found in retrogenes. DinoSL–DinoRL was the most predominant tandem arrange found (6213). (B) DinoSL logo consensus resulting of the alignment of DinoSL/ DinoRL found in retrogenes of all dinoflagellates. Motif length and position are indicated. The height indicates the sequence conservation and the nucleotide frequency in each position. (C) Number of retrogenes, protein-coding retrogenes and percentage of transcriptome assembly completeness (BUSCO) for each dinoflagellate specie. An average of 177 retrogenes per species was identified, and the average assembly completeness was 82%. DinoSL, dinoflagellate-specific splice-leader motif ; BUSCO, benchmarking universal single-copy orthologues.

We took advantage of the presence of a ‘relic’ DinoSL (hereby DinoRL) to identify retrogenes in transcriptome assemblies from dinoflagellates (electronic supplementary material, table S1), which makes the process straightforward and overcomes the general lack of genome sequencing [20,50]. We were particularly interested in studying retrogenes that include the SLTS system as a part of their retroduplication cycle. Therefore, we searched for the DinoSL–DinoRL tandem (37 nucleotides) at the 5′-end of each transcript as the signature for retrogenes (see §2). We retrieved 6544 highly confident retrogenes across 37 of the 43 dinoflagellate species (figure 1C; electronic supplementary material, table S2). DinoSL–DinoRL was the most prevalent arrangement in 95% of the retrogenes (figure 1A). The remaining 5% have at least two DinoRLs tandemly arranged along with DinoSL. This implies that at least 5% of the retrogenes went through multiple recycling events or were subject to two retroduplication events [20]. These findings are consistent with the fact that multiple rounds of retroduplication originated as part of the retrogene repertory of the *Symbiodinium* lineage [21]. Additionally, the DinoSL (figure 1B) consensus is dominated by retrogenes starting with truncated DinoRL (CTCAAG) followed by a DinoRLs (CCATTTTGGCTCAAG), suggesting that the dinucleotide TG may serve as non-canonical 3′ splice acceptor site. We observed this DinoRL pattern in most dinoflagellate orders, except for Noctilucales, where the canonical DinoSL prevailed (electronic supplementary material, figure S1). RNA degradation or epigenetic modification on DinoSL may interfere with capturing the full mRNA structure, potentially underestimating the number of DinoSL and DinoRL, i.e. retrogenes, across dinoflagellates.

Retrogenes were identified in nearly all studied dinoflagellate transcriptomes except for six species (i.e. *Breviolum aenigmaticum, Breviolum psygmophilum, Cladocopium goreaui, Prorocentrum lima, Symbiodinium microadriaticum and Symbiodinium* sp.) most of them from the Suessiales order. This absence could be due to low-frequency retrotransposition or technical limitations in capturing and sequencing complete mRNA molecules. Before this study, retrogenes were identified in seven dinoflagellate genera (i.e. *Symbiodinium*, *Breviolum, Fugacium, Polarella*, *Alexandrium* and *Prorocentrum*) [20,21,51]. Our study expands this to 26 genera (46 dinoflagellate species belonging to 7 taxonomic orders), including dinoflagellates with different lifestyles (i.e. symbiotic and free-living) and genome sizes (approx. 1–220 Gbp) (figure 1C). On average, 177 retrogenes were identified per species (retrogenes isoforms were collapsed within species), with the highest count for *P. lunula* (757) and the lowest for *Oxyrrhis marina* (1). This trend suggests that retroposition is widespread across the dinoflagellate diversity in concordance with the large scale of retroposition predicted for this lineage [20]. Retrogene abundance is generally similar among taxonomic orders, and the most drastic difference is between Gonyaulacales (271 per taxon) and Suessiales (21 per taxon); however, no correlation between retrogene abundance and taxonomic order was detected. Additionally, no correlation between genome size and retrogene abundance was identified partially due to the lack of definitive (sequencing-based) genome size estimations. For instance, *Prorocentrum minimum* has a high number of retrogenes (477) and a relatively medium-low genome size (approx. 4–20 Gbp), whereas *Alexandrium catenella,* with a genome size of around 220 Gbp*,* has 162 retrogenes. The transcriptome assembly completeness was high, and on average, 82% of BUSCO proteins (Aveolata_odb10) for alveolates were identified (figure 1C; electronic supplementary material, table S2), except for *Alexandrium andersonii*, *Alexandrium minutum*, *Cladocopium sp*. and *Durusdinium trenchii* (<50%). As a general observation, transcriptome completeness does not drastically influence the detection of retrogenes across dinoflagellates. For instance, species like *A. minutum* have a high number of retrogenes (696) despite low transcriptome completeness (37%). This suggests that additional factors related to retrogene survival and retrotransposition rate may explain the differential retrogene observed abundance among dinoflagellates. The numbers of this survey confirm that retrogenes and gene retrotransposition are widespread phenomena in the dinoflagellate linage that may imply an ongoing retrotransposon activity or successive bursts leading to rapid functional turnover from ‘dead upon arrival’ retrocopies to *bona fide* retrogenes.

### 3.2. Functional annotation and enrichment

**Figure 2.**
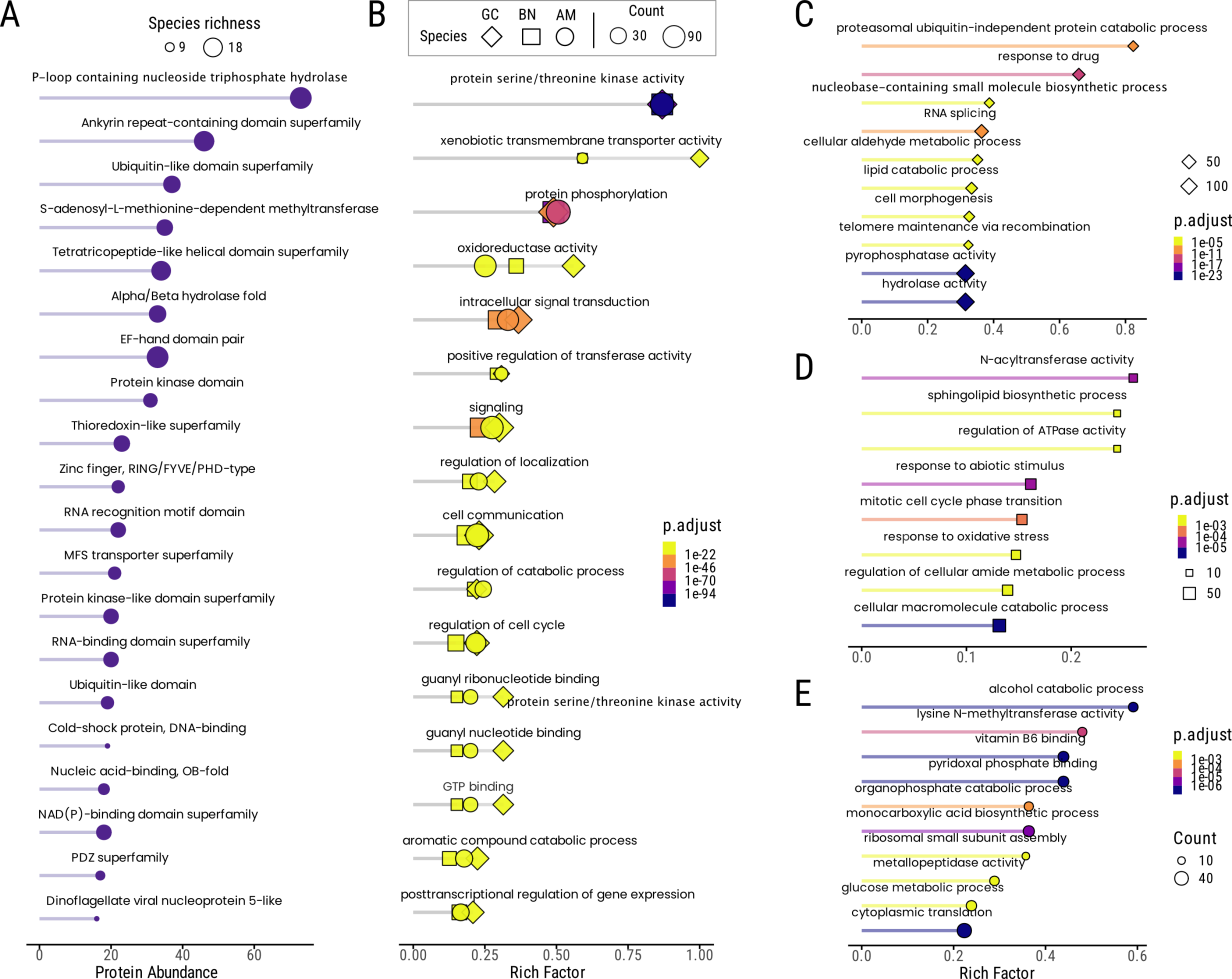
Top 20 most frequent protein domains encoded by retrogenes and enrichment analysis of retrogenes for *Alexandrium molinatum*, *Gymnodinium catenatum* and *Brandtondinium nutricula*. (A) Top 20 InterPro protein domains encoded by retrogene. The size of the circles represents the number of dinoflagellates in which a particular protein domain is present. (B) Sixteen shared enriched GO term for three selected dinoflagellates with the highest retrogene count: *A. molinatum*, *G. catenatum* and *B. nutricula*. Colour scales depict the log_10_ value of *p*-adjusted values, and darker colour intensity indicates higher enrichment. The rich factor is the proportion of retrogenes to genes that are annotated in a particular GO term. Unique enriched GO terms were identified for *G. catenatum* (C), *B. nutricula* (D) and *A. molinatum* (E). GO, Gene Ontology.

A total of 6535 retrogenes were translated to amino acid sequences and comprehensively annotated (NCBI non-redundant, InterPro, PANTHER and PFAM). Out of these, 4742 retrogenes (72%) were successfully annotated to protein families and conserved protein domains (figure 2A; electronic supplementary material, table S6). Some retrogene-encoded proteins may be highly divergent from known homologous proteins, often referred to as dark proteins [52]. Additionally, the unannotated fraction may include pseudogenized retrogenes or short isoforms, as the average peptide size of annotated retrogenes (276 amino acids) was significantly larger than that of non-annotated retrogenes (124 amino acids) (electronic supplementary material, figure S2).

Among the 20 most frequent proteins and domains identified, P-loop NTPase (P-loop containing nucleoside triphosphate hydrolase), ankyrin repeat, ubiquitin, methyltransferases, tetratricopeptide repeats domain, hydrolases, kinases and DNA- and RNA-binding domain or motifs were identified (figure 2A). The presence and frequency of these domains across species underscore both universal and specialized functions, indicating that retrogenes capture evolutionarily conserved gene pathways as well as novel and adaptive roles. For example, domains like P-loop NTPase and EF-hand are frequently found across dinoflagellates and are linked to essential roles and basic cellular functions such as signalling. Conversely, some retrogenes encode protein domains with more restricted distribution, linked to specialized functions like cold-shock proteins and chromatin-associated dinoflagellate/viral nucleoproteins (DVNPs) [53]. This suggests that retrogene diversity is predominantly associated with essential functions (e.g. cell signalling) but also includes less common functions.

We conducted a GO annotation using the PFAM and InterPro predicted domains to understand the functional categorization of our retrogene-coding dataset. About 30–40% of the PFAM and InterPro domains do not affiliate with any GO categories and terms. The GO category ‘biological process’ was the most represented in the retrogene dataset to a lesser degree, ‘cellular component’ and ‘molecular function’, respectively (electronic supplementary material, figure S3). In the ‘biological process’ category, ‘metabolic processing of organic substances’ (e.g. carbohydrates and amino acids), ‘nitrogen, biosynthesis’ and ‘regulation’ are highly represented (electronic supplementary material, figure S3). We conducted an enrichment analysis of the dinoflagellates with the highest retrogene count: *G. catenatum*, *A. monilatum* and *B. nutricula*. These dinoflagellates are representative of different lifestyles (symbiotic: *B. nutricula* and free-living*: G. catenatum* and *A. monilatum*) and are involved in different biological processes such as toxin production, algal bloom and symbiosis. We tested the GO term over-representation for each. We found a core of 16 GO terms shared by the three dinoflagellates (figure 2B). The most significantly enriched terms are related to ‘protein phosphorylation’ and ‘protein serine/threonine kinase activity’ (figure 2B). This indicates that these dinoflagellates have a substantial subset of retrogenes involved in post-translation modifications, in concordance with the heavy reliance of dinoflagellates on this mechanism for gene expression control [27,52]. Consequently, high enrichment of kinases, particularly serine/threonine, was observed (figure 2B). These enzymes play a vital role in dinoflagellate signalling pathways during explosive growth during algal blooms [54]. Other terms enriched among retrogenes are ‘GTP binding’, ‘guanyl nucleotide binding’ and ‘oxidoreductase activity’, which are too ubiquitous to ascribe them to particular roles without additional evidence but are nonetheless compatible with signal transduction and stress response. Subsequently, several enriched terms associated with signalling pathways, such as ‘intracellular signal transduction’, ‘cell communication’, ‘regulation of cell cycle’ and ‘regulation of localization’, were identified (figure 2B). These categories reflect the processing of environmental cues and coordination of cellular responses, potentially leading to increased cell division rates [54]. On the other hand, terms related to ‘aromatic compound catabolic process’ and ‘xenobiotic transmembrane transporter activity’ were enriched (figure 2B), suggesting that dinoflagellates retrogenes are significantly involved in the breakdown of aromatic compounds and the transport of foreign compounds across membranes. The transport of substances across cell membranes has a fundamental role in symbiotic partnership in dinoflagellates such as *B. nutricula* [55] and potentially in the ability to cope with toxic compounds in *G. catenatum* and *A. monilatum*. Overall, the retrogene-enrichment analysis highlighted the functions and pathways involved in post-translational modifications, signalling and regulatory mechanisms that are highly demanded and expressed under stress conditions and in response to environmental triggers.

A number of enriched GO terms pointing to more specific functions or roles were identified for each of the three selected dinoflagellates (figure 2C–E; electronic supplementary material, tables S3–S5). While it is tempting to match some of these categories with certain characteristics of these species, these GO terms usually overlap with many functions. Moreover, functional assignments often rely on the presence of a domain embedded in an otherwise hypothetical protein. In the absence of more detailed information on the individual proteins, any association remain speculative. *G. catenatum* is a toxin producer dinoflagellate characterized for synthesizing saxitoxin and forming toxic algal blooms. Conceivably, some components of the categories enriched in this species may be involved in either self-detoxification (e.g. term ‘response to drugs’) or toxin synthesis (e.g. ‘cellular aldehyde metabolic process’ and ‘lipid catabolic process’) [56,57]. In the case of *B. nutricula*, stress response-associated terms resulted specifically enriched, i.e. ‘response to oxidative stress’ and ‘response to abiotic stimulus’ (figure 2D). The pathways related to these processes (e.g. reactive oxygen species) are highly active under stress conditions like heat increments in symbiotic dinoflagellates [58,59]. Interestingly, the ‘sphingolipid biosynthetic process’ is highly enriched, and it has been described in the context of the symbiosis homeostasis between *Symbiodinium* spp. and its cnidarians host [60]. Overall, retrogenes associated with particular dinoflagellate processes such as toxin production, symbiosis, environmental response and photosynthesis were enriched, highlighting their role in adaptation to particular lifestyles and ecological niches.

We also identified a set of retrogenes encoding proteins for functions overlooked by the enrichment analysis (table 1). These include retrogenes involved in actin regulation (PFN), cell cycle (centrosomal protein) and motility (PIH1). Additionally, a set of less well-known genes and potential candidates for lateral gene transfer were identified, such as membrane-attack complex/perforin (MACPF), nicotinate-nucleotide—dimethylbenzimidazole phosphoribosyltransferase, pectin lyase fold/virulence factor and quinoprotein alcohol dehydrogenase-like superfamily (table 1). This suggests that retrotransposition may also target genes that are not part of the massive functional core but are environmentally triggered and potentially less transcriptionally active.

**Table 1.** Retrogene encoding proteins associated with common cellular processes in *Gymnodinium catenatum*, *Alexandrium monilatum* and *Brandtondinium nutricula*.

accession no	description	abbreviation	dinoflagellate
toxin metabolism			
PTHR43900	glutathione s-transferase	*GST*	*Gymnodinium catenatum*
PTHR20961	glycosyltransferase	*Glycosyltransferase_61*	*G. catenatum*
PTHR43625	aldo/keto reductase	*Aldo-Keto_reductase*	*G. catenatum*
IPR003582	shk domain-like	*ShKT_dom*	*Alexandrium monilatum*
IPR030834	polyketide synthase-associated domain	*PKS_assoc_dom*	*A. monilatum*
PTHR46701	glycosyltransferase-like kobito 1	*KOBITO1-like*	*A. monilatum*
photosynthesis			
PTHR34812	photosystem ii reaction center protein j	*PSII_PsbJ*	*G. catenatum*
PTHR34688	cytochrome c6, chloroplastic	*Cyt_C6*	*G. catenatum*
PTHR34264	ATP synthase subunit b, chloroplastic	*ATP_synthase_F0_b*	*G. catenatum*
PTHR42823	ATP synthase subunit a, chloroplastic	*ATP_synthase_F0_ba*	*A. monilatum*
PTHR13822	ATP synthase epsilon chain, chloroplastic	*ATP_synth_F1_dsu*	*A. monilatum*
PTHR34469	photosystem ii reaction center protein h	*PSII_PsbH*	*A. monilatum*
PTHR35325:SF1	photosystem ii reaction center protein k	*PSII_PsbK*	*A. monilatum*
PTHR34058	oxygen-evolving enhancer protein 1−2, chloroplastic	*PsbO*	*A. monilatum*
PTHR42864	light-independent protochlorophyllide reductase iron-sulfur ATP-binding protein	*Protochlorophyllide_ATP-bd*	*G. catenatum*
environmental response			
PTHR11709	multicopper oxidase	*Cu-oxidase*	*G. catenatum*
PTHR24291	cytochrome p450 family 4	*Cytochrome_P450_Monoox*	*Brandtodinium nutricula*
PTHR43057	arsenite efflux transporter	*Arsenical-R_Acr3*	*B. nutricula*
PTHR13748	COBW-related	*Zinc-reg_GTPase_activator*	*B. nutricula*
PTHR23248:SF9	phospholipid scramblase	*Scramblase*	*B. nutricula*
carbohydrates metabolism			
PTHR11183	glycogenin subfamily member	*GNT1/Glycosyltrans_8*	*B. nutricula*
IPR001722	glycoside hydrolase	*Glyco_hydro_7*	*B. nutricula*
IPR049892	hypothetical endoglucanase	—	*B. nutricula*
others			
PTHR45742	membrane attack complex	*aMACPF*	*B. nutricula*
PTHR11604:SF0	profilin	*PFN*	*B. nutricula*
PTHR23159	centrosomal protein 2	*Centro_Cilia_Struct_Component*	*G. catenatum*
PTHR22997:SF3	protein kintoun; motility	*PIH1*	*G. catenatum*
PTHR43463	nicotinate-nucleotide–dimethylbenzimidazole phosphoribosyltransferase	*aNict_dMeBzImd_PRibTrfase*	*A. monilatum*
IPR011050	pectin lyase fold/virulence factor	*aPectin_lyase_fold/virulence*	*A. monilatum*
IPR011047	quinoprotein alcohol dehydrogenase-like superfamily	*aQuinoprotein_ADH-like_supfam*	*A. monilatum*

^a^ Potential bacterial viral origin

### 3.3. Retrogenes expression and codon usage trend

**Figure 3.**
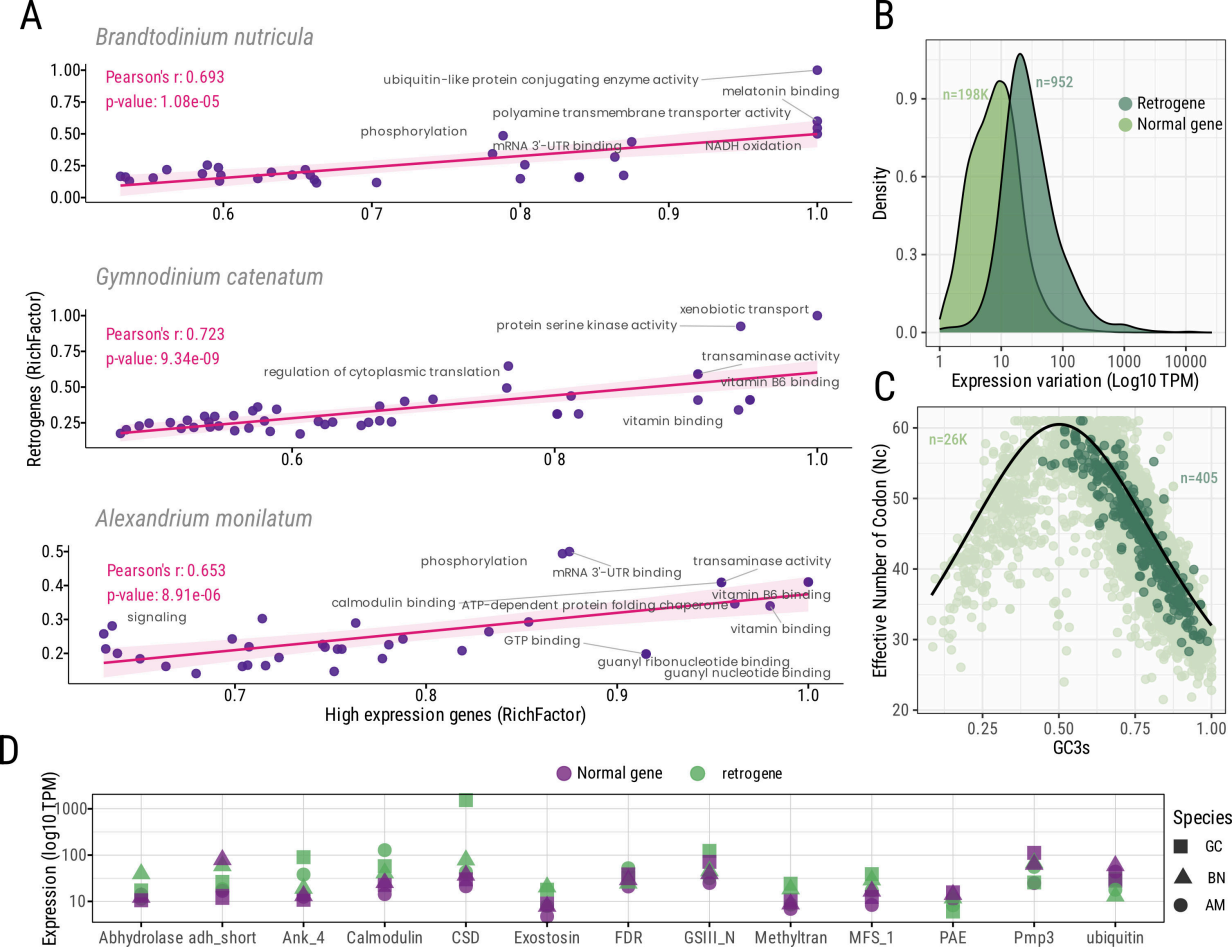
Retrogene expression and codon usage analysis. (A) correlation of enriched GO categories between retrogenes and genes with high expression profile. (B) Density distribution of the gene expression (log_10_ TPM) for retrogenes (dark green) and ‘normal genes’ (light green). (C) Effective number of codon (Nc) relative to the GC percentage at synonymous third codon position (GC3s). The solid black curve represents the expected values for random codon usage. (D) Expression of shared retrogenes associated with most enriched functions compared with average expression of ‘normal gene’. Gene abbreviations: MFS1, major facilitator superfamily 1; AB hydrolase, alpha/beta hydrolase superfamily; Pae, pectin acetylesterase; Hypoth. FRD, hypothetical fumarate reductase; Adh_short, dehydrogenases, short-chain; Pmp3, proteolipid membrane potential modulator; GSIII, glutamine synthetase; Ank, ankyrin repeat protein; Csd, cold-shock domain protein; GO, Gene Ontology.

We compared the enrichment of highly expressed genes that overlapped with the expression pattern of retrogenes (figure 3B). We found that approximately 40–50% of the enriched GO categories were shared, and the enrichment magnitude of each directly correlates (figure 3A; electronic supplementary material, table S8). This suggests that retrogenes are likely derived from genes with high expression profiles involved in cell signalling, transport and gene expression regulation (figure 3A). We analysed and compared the expression of 13 individual retrogenes shared by *G. catenatum*, *A. monilatum* and *B. nutricula* as representatives of the shared core of functions associated with environmental response, transport and cold adaptation (figure 3D). Retrogenes generally showed equal or higher expression levels compared with ‘normal genes’, particularly in *G. catenatum* and *B. nutricula* (figure 3B; electronic supplementary material, figure S4), indicating a greater reliance on retrogenes for certain cellular functions in these dinoflagellates. For instance, the cold-shock domain (Cds) and calmodulin exhibited higher expression levels in *A. monilatum* and *G. catenatum*, respectively. Additionally, retrogenes involved in metabolic function and transport—such as the MFS transporter superfamily (MFS1), Ab-hydrolase, methyltransferase-21 and exostosin—showed increased expression in *G. catenatum* and *B. nutricula*. Likewise, glutamine synthetase (GSIII_N) and ankyrin repeat (Ank) resulted highly expressed in *G. catenatum* retrogenes. The high expression levels detected for some retrogenes suggest their potential role in significantly boosting the expression level and protein abundance. Retrogenes related to stress response and metabolism appear to be particularly upregulated, potentially aiding dinoflagellates in adapting to their environments and maintaining cellular processes. To determine if retrogene formation is largely neutral (transcript abundance driven) or there is a component of selection favouring higher rates of retrotransposition, we attempted to examine the patterns of diversification of retrogenes among *Alexandrium* species (best taxon sampling and retrogene counts). However, very few homologous retrogenes overlap between more than two species, and the ones that do encode short proteins with little phylogenetic signal.

We compared the transcriptional landscape of retrogenes and ‘normal genes’ across three selected dinoflagellates (figure 3B,C). The operational status of retrogenes was evaluated by analysing their expression profiles and codon usage bias, given that expression levels typically correlate negatively with codon usage bias [61]. Retrogene expression patterns were consistently higher in all three dinoflagellates (figure 3B, electronic supplementary material, figure S7). While not statistically significant, these differences suggest that retrogenes recapitulate the expression pattern from their parental genes. For codon usage analysis, we compared over 25 000 ‘normal gene’ CDSs sequences (complete CDSs) with approximately 400 retrogene CDS sequences across the three dinoflagellates (figure 3C; electronic supplementary material, figure S4). The effective number of codons (Nc) was similar for both gene categories, averaging 44.5 for ‘normal genes’ and 45.1 for retrogenes (electronic supplementary material, table S8). A small fraction of both categories had Nc < 35 (1740 ‘normal genes’ and 34 retrogenes), indicating marginal codon bias. Likewise, the GC content was similar between the two gene categories (62.7% for ‘normal genes’ and 61.3% for retrogenes) (electronic supplementary material, table S7). High GC content at the third codon position (GC3s) was identified in both gene categories, but no significant difference in the GC3s was detected (electronic supplementary material, table S8). We plotted Nc versus GC3s for ‘normal genes’ and retrogenes CDSs along with the standard curve. We found values clustered between 25−60 and 30−60 for coding sequence and retrogenes, respectively (figure 3C). Overall, retrogenes distribution mirrors the general trend of ‘normal genes’, showing a high GC-biased codon usage for the three dinoflagellates. GC content is particularly high in the protein-coding fraction in most dinoflagellates (>60%) [33,62,63], indicating that little margin is left for codon optimization in retrogenes since around 75% of GC3s were detected in concordance with previous findings [63].

## 4. Discussion

Retroposition generates new gene copies from transcribed genes, contributing to genome evolution by directly or indirectly participating in gene birth-and-death processes, genome size dynamics and adaptation. Dinoflagellates are an ideal lineage to study retroposition due to the advantage of DinoRL as the hallmark of the retrogenes [20,50]. Here, we conducted a comprehensive survey of retrogenes in all available dinoflagellate transcriptomes, focusing on understanding their functional categorization, distribution and operational status.

The actual number of retrogenes is likely severely underestimated due to partial or incomplete 5′ ends. Technical limitations of RNA-seq, RNA degradation and epigenetic modifications may also hinder the detection of retrogenes. For instance, between 0.68 and 50% of full-length transcripts containing DinoSL were detected in *Polarella glacialis* using the full-length transcript sequencing method (PacBio IsoSeq). Still, less than 1% exhibited the DinoSL–DinoRL motif [51]. This discrepancy is likely due to a double methylcytosine in DinoSL, causing 5′-end truncations during library preparation. Additionally, degeneration of DinoSL–DinoRL tandems beyond detection would further underestimate retrogene detection [19,20]. Despite these challenges, retrogenes were identified across most dinoflagellate orders, aligning with initial estimates based on a pool of EST data from various dinoflagellates [20]. This suggests that retrogene formation or gene retroduplication is a continuously active process in dinoflagellates, likely mediated by retrotransposons. Besides mRNA degradation and epigenetic modification, additional factors may influence retrogene abundance across dinoflagellates, such as the size of gene repertoire, retrotransposon activity and regulation and selective constrictions for genome size.

### 4.1. DinoSL functional association and retrogenes

SLTS is a distinctive phenomenon in dinoflagellates, widespread in all nuclear mRNA. Recent studies suggest that this process may predominantly target specific functional categories within the transcriptome of dinoflagellates and *Perkinsus* [51,64]. An interesting case of SL variants has been reported for *Perkinsus,* where different SL-types segregate the transcriptome into various functional categories, enhancing transcript abundance [64]. While the exact mechanism of how SL-types segregate transcriptomes remains unknown, they may influence translation efficiency. In dinoflagellates, it is unclear how functionally related sets of transcripts are highly expressed and enriched with the same DinoSL [51]. We identified a highly confident set of retrogenes with a conserved DinoSL sequence among dinoflagellates, with no additional variants found. These retrogenes were preferentially associated with stress response and cell signalling functions, as well as retrogenes encoding for specific functions such as cold-shock protein, DVNPs and MACPF. The SLTS process appears essentially random, and its association with functionally related genes may reflect genome organization and transcription patterns. On one hand, abundant transcripts are more likely to provide substrate for reverse transcription and subsequent integration as potential retrogenes. Thus, functional categories with high expression profiles will tend to be more represented by retrogenes. On the other hand, functionally related genes, such as those involved in photosynthesis and stress response, are often located in close proximity within the genome, usually forming tandem arrays that act as transcriptional units [65,66]. This proximity might result in DinoSL genes being transcribed simultaneously with their target genes, leading to high expression, functionally related genes sharing DinoSL. Gene retroposition may favour this functional association by integrating retrocopies near the parental copies. However, the available data are not sufficiently abundant to allow for a definitive testing to favour one scenario (i.e. highly expressed genes are more likely to be retroposed simply due to higher transcript availability) versus the other (i.e. natural selection drives the diversification of functionally important genes through retroposition). Such testing requires considerable overlap of homologous retrogenes across multiple species, but this only happens sporadically in the current dataset. Further research is needed to understand the SLTS system in dinoflagellates and the relationship between the retroposition mechanisms and rates and the functional implications.

### 4.2. Post-translational modification pathways emerge as prominent features among retrogenes

We aimed to determine whether the set of retrogenes is enriched in any particular functional categories. We contrasted the retrogenes of three dinoflagellates belonging to three different orders (Gymnodiniales, Peridiniales and Gonyaulacales) with different genome sizes and lifestyles. Our analysis revealed a core set of highly enriched GO terms associated with stress response and cell signalling functions. Prominent pathways included post-translational modification (e.g. protein phosphorylation, serine/threonine kinase activity), cell signalling (e.g. intracellular signal transduction and calmodulin binding) and transport (e.g. xenobiotic transport). These processes were highlighted by the abundance of retrogene-encoded protein domains, such as ion transport, protein kinase, calmodulin, GTP-binding proteins and methyltransferases. Additionally, each dinoflagellate species exhibited specific enriched terms related to their unique biological traits, such as toxin production, symbiosis and photosynthesis. This underscores the role of retrogenes in contributing to key biological processes and adaptations while also maintaining essential functions like cell signalling.

Regarding the mechanism of retrogene formation, stress conditions such as high temperatures, light intensity and nutrient richness result in algal blooms and are shown to activate both transposable and viral elements in dinoflagellates [21,67,68]. The retroposition mechanism invokes the retrotransposon’s off-target activity in reverse transcriptase activity on cellular transcripts [3,4]. Both LTR and non-LTR retrotransposons were found highly abundant in dinoflagellates genomes [26,51], in some cases active [69]. Transcriptional repression of retrotransposons can be weak during stress periods, leading to a burst of retrotransposon activity. It is not clear whether retrotransposon activity is ongoing or happens in specific periods. Much of the epigenetic regulation of these elements is unknown, although the modified base 5HmU might play a role [65,70]. Gene retroduplication likely involves synchronization between gene expression levels and retrotransposon activity, with highly expressed genes being more prone to retroposition [24]. In this sense, stress-related gene functions involving cell signalling and photosynthesis highly preponderant in toxic dinoflagellates blooms [71–74] are more likely to become retrogenes. On the other hand, retrogene expression profiles may mimic their parental gene pattern (i.e. similar tendency of codon bias and expression pattern distribution), potentially reinforcing or slightly modifying their original functions. In fact, most of the retrogenes in *Symbiodinium* are ‘orphans’ with no parental gene found, and it has been shown that orphan retrogenes can recapitulate the expression pattern of the parent gene in mammals [75]. Understanding how retrogenes become active and acquire the already scarce regulatory sequences is intriguing. It could be that sequences that can drive transcription (even if at low levels) are ubiquitous, increasing the likelihood of new retrotranscribed sequences being expressed. Moreover, a TTTT box found in the DinoSL could itself be able to promote transcription [21]. Therefore, full-length retrocopies carry their own basal promoter. Additionally, transcripts that went through several turns of recycling recently will carry more relics, enhancing the chances of being expressed upon integration.

## 5. Conclusion

This retrogenes survey provided evidence for widespread retrogenes among dinoflagellates, highlighting post-translational prominent pathways and close association with stress response and cell signalling. The study also highlights the preponderance of retrogenes associated with unique biological traits. The synchronization between gene expression and retrotransposon activity may play a key role in the gene retroposition process. The retrogene expression pattern may mirror those of their parental gene, suggesting some functional integration. The mechanism underlying retrogene activation and regulation remains unknown and requires additional investigation with more comprehensive data collection. Understanding these processes can provide deeper insights into the evolutionary and adaptive significance of retrogenes in dinoflagellates and other organisms.

## Data Availability

Supplementary material is available online [76].
